# The Long and Thorny Road to Publication in Quality Journals

**DOI:** 10.1371/journal.pcbi.0030251

**Published:** 2007-12-28

**Authors:** Thomas C Erren

Within the “Ten Simple Rules” series in *PLoS Computational Biology*, Dr. Bourne suggests that for younger investigators it is better to publish one paper in a quality journal rather than having multiple papers in lesser journals [1]. While this is certainly advisable, it can be very difficult. Indeed, for young scientists or, more to the point, for researchers with a short record of publications, it may be almost impossible to make their work and themselves visible to a larger scientific community via higher impact journals. A not-too-small share of “seasoned” scientists will argue without malignity that “we experienced similar or the same” and “good researchers will eventually be recognized.” What they imply is that those who continue to provide good science shall be rewarded later, i.e., their papers will eventually find a home in quality journals, thus yielding better chances that the work will have impact. And yet, a much-cited case study ([2]; cited 264 times as of November 18, 2007, according to http://isiwebofknowledge.com/) may illustrate that the road to publication and recognition can be thorny and long for younger and less-recognized scientists.

Indeed, this “experiment” by Peters and Ceci provided empirical evidence 25 years ago that to get a paper accepted for publication can be very difficult for lesser-known scientists from less-recognized institutions. In this study, 12 psychology articles that had already been published by prestigious scientists from prestigious institutions were resubmitted to the journals that had accepted and printed the papers in the first place. Data presentation remained almost unaltered, but fictitious names and not-well-known institutions replaced the original ones. Only three of the resubmissions were identified as such, and of the other nine manuscripts, eight were rejected, mainly for methodological reasons. The Peters and Ceci study was widely discussed, and one interpretation for their observations was that work from lesser-known researchers may be subjected to a more critical peer review than material submitted by well-known investigators in institutions with a long track record. To exemplify this notion, 1977 Nobel Laureate Rosalyn Yalow commented on the article by Peters and Ceci “. . . . I am in full sympathy with rejecting papers from unknown authors working in unknown institutions. How does one know that the data are not fabricated? . . . on the average, the work of established investigators in good institutions is more likely to have had prior review from competent peers and associates even before reaching the journal” [3].

Despite this background, Dr. Bourne is right when he suggests that young investigators should aim at publication in quality journals. After all, you can only score high if you try. But be prepared that it takes very good material and perseverance to publish in well-known journals. Be aware, also, that even the highest-quality work may not see publication in high-impact journals, for numerous reasons, with the novice status of the submitting author(s) likely being a primary one. In this vein, both less and more experienced researchers may want to read the following paper for empirical comfort: “Consolation for the scientist: Sometimes it is hard to publish papers that are later highly cited” [4]. 
